# Enhanced Endothelin-1 Mediated Vasoconstriction of the Ophthalmic Artery May Exacerbate Retinal Damage after Transient Global Cerebral Ischemia in Rat

**DOI:** 10.1371/journal.pone.0157669

**Published:** 2016-06-20

**Authors:** Frank W. Blixt, Sara Ellinor Johansson, Leif Johnson, Kristian Agmund Haanes, Karin Warfvinge, Lars Edvinsson

**Affiliations:** 1 Department of Clinical Sciences, Division of Experimental Vascular Research, Lund University, Lund, Sweden; 2 Department of Clinical Experimental Research, Glostrup Research Institute, Rigshospitalet, Glostrup, Denmark; 3 Department of Ophthalmology, Glostrup Research Institute, Rigshospitalet, Glostrup, Denmark; University of Florida, UNITED STATES

## Abstract

Cerebral vasculature is often the target of stroke studies. However, the vasculature supplying the eye might also be affected by ischemia. The aim of the present study was to investigate if the transient global cerebral ischemia (GCI) enhances vascular effect of endothelin-1 (ET-1) and 5-hydroxytryptamine/serotonin (5-HT) on the ophthalmic artery in rats, leading to delayed retinal damage. This was preformed using myography on the ophthalmic artery, coupled with immunohistochemistry and electroretinogram (ERG) to assess the ischemic consequences on the retina. Results showed a significant increase of ET-1 mediated vasoconstriction at 48 hours post ischemia. The retina did not exhibit any morphological changes throughout the study. However, we found an increase of GFAP and vimentin expression at 72 hours and 7 days after ischemia, indicating Müller cell mediated gliosis. ERG revealed significantly decreased function at 72 hours, but recovered almost completely after 7 days. In conclusion, we propose that the increased contractile response via ET-1 receptors in the ophthalmic artery after 48 hours may elicit negative retinal consequences due to a second ischemic period. This may exacerbate retinal damage after ischemia as illustrated by the decreased retinal function and Müller cell activation. The ophthalmic artery and ET-1 mediated vasoconstriction may be a valid and novel therapeutic target after longer periods of ischemic insults.

## Introduction

Global cerebral ischemia (GCI) occurs when blood supply is significantly reduced to the entire brain, suddenly leaving the affected tissues severely deprived of oxygen and glucose. In clinical conditions, the most common source of global cerebral ischemia is cardiac arrest, a devastating and common cause of death in the western world [1, 2]. Furthermore, ocular blood supply originates from the common and internal carotid arteries. Thus a carotid artery occlusion can lead to delayed ocular complication such as ocular ischemic syndrome as reviewed by Terelak-Borys and colleagues [3], thus global ischemia has an effect on the eye.

Recent data suggests that a key factor in ischemic pathophysiology is the regulation of vascular contraction. Endothelin-1 (ET-1) is a highly potent vasoconstrictor and its effect in cerebrovascular ischemic conditions is well described [4]. ET-1 acts on two main receptors, ET_A_ and ET_B_. ET_A_ is largely found in smooth muscle cells of vasculature inducing strong vasoconstriction. ET_B_ function, however, is slightly more intricate. Under normal physiological conditions, ET_B_ is mainly found on the vascular endothelial cells, propagating NO release, which acts as a vasodilator agent [5]. Nonetheless, in certain conditions ET_B_ receptors are found to be upregulated and expressed on the vascular smooth muscle cells, causing strong vasoconstriction [6].

The brain and the eye share a common embryological origin, but very little has been done to investigate whether the ocular vasculature responds in a fashion similar to cerebral vasculature during ischemic conditions [6]. The ophthalmic artery, the key supplier of blood to the retina and eye, can potentially play an important role in ocular ischemic pathophysiology. Notably, active ET-1 receptors have been found on the ophthalmic arteries of humans [7] and rat [8]. Additionally, glial cell activation in the retina is a clear indicator whether homeostatic blood flow has been altered to the eye.

Müller cells are the chief glial cells of the retina and span from the inner limiting membrane to the outer limiting membrane. When activated after an ischemic insult they initiate gliosis, a process which attempts to re-establish homeostatic conditions in the retina [9]. The most efficient way of monitoring Müller cell activity is via glial fibrillary acidic protein (GFAP) [10] and vimentin [11] expression.

However, activation of Müller cells does not give any insight on whether vision has been compromised. To this end, electroretinography (ERG) can be used for the evaluation of functional changes to the retina. Using light stimuli, ERG results in a photoreceptor dependent a-wave, an ON-bipolar cell dependent b-wave, inner retinal dependent oscillatory potentials (OPs), and a (primarily) ganglion cell dependent scotopic threshold response (STR) [12]. By combining immunohistochemistry and ERG we can assess the severity of damage caused to the retina during global cerebral ischemia.

Retinal ischemia has been investigated before and one earlier study by Zhao and colleagues, did not observe any retinal changes 48 hours following 17 minutes of global ischemia. However, in our previous studies we have shown that using a 15 minute global cerebral ischemia model, an up-regulation of the 5-hydroxytryptamine/serotonin receptor, 5HT1B, and the ETB receptor occurs in the rat middle cerebral artery (MCA) and anterior cerebral artery (ACA) 48 hours after ischemia and reperfusion. The cerebral blood flow has been monitored in this method has been verified through MRI and laser Doppler [13, 14]. We therefore postulated that there might be a delayed ischemic damage caused by an upregulation of contractile receptors in the vasculature, and that significant retinal damage can be delayed. It follows that increased contractility to ET-1 leads to decreased blood flow as seen in the cerebral vasculature [7], where vascular receptor changes further decrease blood flow and exacerbates damage to the surrounding brain tissue after the original ischemic insult.

Therefore, we examined the contractility of the ophthalmic artery after ischemia and monitored ERG alterations occurring in parallel with activation of Müller cells at the earlier time points as well as at both 72 hours and 7 days after ischemia. We show that although the retina is resistant to ischemic damage, activation of Müller cells only occur in the retina after we observe an increased contractility in the ophthalmic artery. We therefore propose that changes in the ophthalmic artery are an important factor to fully understand retinal ischemia, particularly for the delayed damage.

## Material and Method

### Animals

All animal experiments were approved under the license number 2012-15-2934-726 and were housed and treated under the strict national laws and guidelines issued by the Danish Animal Experimentation Incorporate. Rats were housed under 12 hour light dark cycle condition and fed standard rat chow with free access to water.

### Global Cerebral Ischemia

Male Wistar rats weighing 260–380g were used for the experiment (Table 1). Prior to the operation the rats were fasted overnight with free access to water.

**Table 1:** Total number or rats used (A) and group allocation (B).

**A. Total Rats Operated**			
	*Total*	*Ischemic*	*Sham*
No. of rats	36	16	15
**B. Distribution of Rats in the Various Experimental Groups**			
Myograph	12	6	6
Immunohistochemistry	24*	14	10
ERG	12	6	6

The total number of rats operated for this experiment.

*Rats used for immunohistochemistry included 4 ischemic and 4 sham operated rats from both the myograph and the ERG group.

Reversible global cerebral ischemia was induced by a 15-minute two vessel carotid artery occlusion couple with simultaneous hypovolemia as previously described [14, 15]. In short, rats were anesthetized with 4% isoflurane (Abbott Laboratories) in N_2_O/O_2_ (70:30), then intubated and artificially ventilated with 1.5%-2% isoflurane in N_2_O/O_2_ (70:30). The tail artery and vein were used for blood pressure recording, regular blood sampling for gas analysis (Radiometer, Copenhagen, Denmark), and infusions, via inserted catheters. Both body and head temperature was monitored at all times during the surgery with a rectal and skull thermometer respectively, and the rat was kept on a heating pad set for 37°C for the duration of the operation. A heparinized soft poly urethane catheter was inserted via the external jugular vein towards the right atrium. Temporary ligatures were placed around both common carotid arteries. When the preparations were completed the rat received 0.5 ml heparin (100 IU/ml) and was equilibrated for 15–20 minutes.

After equilibration, reversible ischemia was induced by lowering the mean arterial blood pressure (MABP) to 40 mmHg by withdrawing blood through the jugular vein catheter, followed immediately by bilateral common carotid artery clamping. After 15 minutes, the clamps were released and the MABP was restored. Sodium bicarbonate (0.4 ml 0.6M) was injected additionally to counteract systemic acidosis. The rat was allowed to recover for 15–20 minutes after all catheters were removed and all incisions were closed before discontinuing isoflurane and extubation. Sham operated animals underwent the same procedure with the omission of bilateral common carotid clamping, lowering of MABP, and sodium bicarbonate injection. Rats were then kept alive for 24 hours, 48 hours, 72 hours, and 7 days.

### Myography

Wistar rats were anesthetized using CO_2_ and decapitated. The eyes were gently removed and the ophthalmic artery dissected out in Na-Krebs buffer containing: NaCl 119 mM, NaHCO_3_ 15 mM, KCl 4.6 mM, MgCl_2_ 1.2 mM, NaH_2_PO_4_ 1.2 mM, CaCl_2_ 1.5 mM and glucose 5.5 mM. The ophthalmic artery segments (1–2 mm) were mounted on a pair of metal wires (25 μm) in an arterial myograph. One wire was attached to a micrometer screw which allows for fine adjustments of the distance between the wires, controlling the vascular tone. The second wire was connected to a force displacement transducer paired together with an analogue-digital converter (AD Instruments, Oxford, UK). All data was recorded on a computer with PowerLab unit software (AD Instruments).

Aerated bicarbonate buffer, composed of 95% O_2_ and 5% CO_2_, with a resulting pH of 7.4, was heated to 37°C and used to immerse the ophthalmic artery segments into the myograph. The segment was normalized to reach 90% of the internal circumferences that a fully relaxed vessel would have under a transmural pressure of 50 mmHg. A benchmark value of the contractile capacity was achieved by temporarily replacing part of the NaCl in the buffer solution with 60 mM K^+^. Artery segments were omitted from the study if they failed to fulfill the inclusion criteria: a maximum contractile capacity of at least 0.5 mN.

All contractile responses are expressed as percentage of the average maximal contraction induced by the K^+^ response. The concentration response curves were made in a cumulative manner for the ET-1 (PolyPeptide Group, Sweden) and 5-carboxamidotryptamine (5-CT, a 5-HT analog; Sigma Aldrich, Germany) agonists.

### Tissue Preparation

Prior to dissection, eyes were marked at the inner angle with a diathermy burner (FIAB SpA, Italy). This allowed for consistent orientation when embedding and cryo-sectioning. The eyes were carefully dissected out and briefly put in 4% formaldehyde in phosphate buffer saline (PBS) to stabilize, allowing for the removal of the cornea and lens. Thereafter, the eyes were re-immersed in 4% formaldehyde in PBS for 3 hours. Next, the eyes were washed in rising concentration from 10% to 25% of sucrose in Sörensen’s phosphate buffer (pH 7.2) for cryo-protection. Finally the eyes were embedded in a gelatin medium containing chicken egg albumin, and positioned according to the burned marking allowing for vertical cryostat sectioning, and stored at -20°C. Eyes were cut in vertical sections through the optic nerve for histology and immunohistochemistry.

### Histochemistry

10 μm cryosections were stained with hematoxylin and eosin to evaluate the morphology of the dissected eyes prior to immunohistochemical evaluations. The slides were submerged in fresh hematoxylin for 1.5 minutes before rinsed in distilled water and washed in tap water for 5 minutes. After a brief submersion in fresh eosin for 1 minute, gradual dehydration was performed in rising concentrations of ethanol before the slides were immersed in xylene. Finally, they were mounted with Pertex mounting medium (Histolab, Gothenburg, Sweden).

### Immunohistochemistry

Cryosections were washed in PBS with 0.25% Triton (PBS-T) for 15 minutes. Next, antibodies against GFAP (rabbit, 1:1500) and vimentin (mouse, 1:400) were applied (for details, see Table 2). The sections were then incubated overnight in incubation chambers at +8°C. The following day, the slides were washed 2x15 minutes in PBS-T followed by the application of appropriate secondary antibodies. Slides were incubated for one hour at room temperature before washing 3x15 minutes with PBS-T and mounted with Vectashield mounting medium containing DAPI (Vector Laboratories, Burlingame CA, USA).

**Table 2:** Full list of antibodies used.

**A. Primary Antibodies**						
*Name*	*Host*	*Dilution*	*Source*	*Cat #*	*Antigen*	*Antibody Registry #*
GFAP	Mouse	1:400	Millipore, Billerica, MA, USA	IF03L	GFAP	AB_212974
GFAP	Rabbit	1:1500	Dako Cytomation, Denmark	Z0334	GFAP in cow spinal cord	AB_10013482
Vimentin	Mouse	1:400	Sigma Aldrich, St Louis, MO, USA	V6630	Vimentin in pig eye lens	V6630
**B. Secondary Antibodies**						
*Name*	*Host*	*Dilution*	*Source*	*Cat #*		
Alexa 594 anti-mouse	Goat	1:400	Invitrogen, Carlsbad, CA, USA	A-11032		
FITC anti-mouse	Donkey	1:200	Jacksson Immunoresearch, West Grove, PA, USA	735-095-151		
Alexa 594 anti-rabbit	Donkey	1:400	Jacksson Immunoresearch, West Grove, PA, USA	711-585-152		

The full list of primary (A) and secondary (B) antibodies used during immunohistochemistry, including dilution, host animal, and manufacturer.

For double staining, the staining was done sequentially. Considerations were made in selecting antibodies so that their host species would not conflict with each other. Each set of immunohistochemical staining was performed a minimum of three times, each coupled with a negative control where the primary antibody was omitted. A full list of antibodies used is found in Table 2.

### Microscopy and Imaging

The slides were examined using an epifluorescence microscope (Nikon 80i, Tokyo, Japan) combined with a Nikon DS-2MV camera. Areas of interest were photographed with 10x, 20x, or 40x lenses. The images were then processed using Adobe Photoshop CS3 (v10.0 Adobe Systems, Mountain View, CA) and images taken with different wavelength filters were superimposed over each other to determine any potential co-localization.

### Electroretinography

Rats were dark adapted overnight and then prepared for ERG recordings under dim red light. Each rat underwent three measurements, 24 hours before ischemia, 72 hours after, and 7 days after ischemia. The rats were anesthetized in accordance with previous studies [16] using ketamine (85mg/kg) and xylazine (20 mg/kg) to allow for comparisons with previously published ERG data. The rat was then placed on a heated platform to maintain body temperature at approximately 37°C. Next, one drop each of oxybuprocain (0.4%), tropicamide (1%) and phenylephrine (5%) were added to each eye for pupil dilation and topical anesthesia. A reference electrode was inserted into the mouth of the rat, gold ring electrodes were positioned on the corneas of the eyes and a subcutaneous needle in the tail served as a ground. ERGs were recorded using a Ganzfield bowl equipped with both LED and xenon lamps (model Q450 SCX, Roland consult, Siegburg, Germany) and digitized at 2.5 KHz over a 400 msec interval with Viking Select analysis software (Nicolet Biomedical Instruments, Madison, WI, USA). Scotopic threshold responses (STR) were elicited with a stimulus of -5.8 log cd*s/m^2^ and 20 responses were averaged, which was sufficient to elicit both a positive and negative STR. ERGs were then recorded for stimuli of -3.7, -3.0, -2.0, -1.0, 0.0, 1.0, 1.5, 1.75 and 2.0 log cd*s/m^2^. The interstimulus interval for intensities of -1.0 log cd*s/m^2^ and less was 5 seconds while for intensities above -1.0 log cd*s/m^2^ it was increased incrementally up to 110 seconds. For the lower intensities, ten responses were averaged, while for the higher intensities the average was made from 2–7 responses.

The ERG trace raw data were exported into Microsoft Office Excel (2003). It has been shown that a delayed Gaussian function can be used to model the leading edge of the a-wave (P3) [17] as described by Hood and Birch [18]. A modification of this description has been used [19] to model P3 amplitude as a function of luminous energy (*i*, log cd*s/m^2^) and time (*t*, seconds):

$$P3\left(i,t\right)=Rm_{P3}[1-\text{exp}\left(-i*S*\left(t-t_{d}{)}^{2}\right)\right],\;t>t_{d}$$

where *Rm*_*P3*_ is the saturated response amplitude, *S* (log m^2^*cd^-1^*s^-3^) is sensitivity reflecting amplification of the phototransduction processes and *t*_*d*_ (seconds) is a delay in time due to the inherent lag of recording equipment as well as physiological processes involved in the photoreceptor response. In this study, this equation was used to model recorded responses to luminous energies of 1.5, 1.75 and 2.0 log cd*s/m^2^. Minimization of the sum of square merit function was used to optimize the parameters *Rm*_*P3*_ and *S* with the solver module of Excel, while *t*_*d*_ was fixed at 3.6 ms.

The amplitude of the b-wave (*V*, μV) can also be modelled as a function of luminous energy (*i*, log cd*s/m^2^) of the stimulus using the Naka-Rushton equation:

$$V\left(i\right)=V_{max}*\frac{i}{i+k}$$

where *Vmax* (μV) is the saturated response amplitude and the semi-saturation constant *k* (log cd*s/m^2^) is the luminous energy required for a half maximal response. Minimization of the sum of square merit function was used to optimize the parameters *Vmax* and *k* with the solver module of Excel. Plotting b-wave amplitude versus energy resulted in a two branches, most likely due to cone contribution at higher energies, and so responses up to and including -2.0 log cd*s/m^2^ and responses above this energy were modelled independently of one another [20, 21].

OPs were extracted from scotopic responses to a stimulus of 2.0 log cd*s/m2 with a band-pass filter of 75–300 Hz by using an add-in function for Microsoft Office Excel (www.web-reg.de/bp_addin.html#).

After ERG recording and while the rats were still anesthetized, fundus images were taken using the Micron IV (Phoenix Research Laboratories, Pleasanton, CA, USA) retinal imaging system and StreamPix software (NorPix, Inc., Montreal, QC, Canada).

## Results

### Global Cerebral Ischemia

The mortality rate of the global cerebral ischemia operation was 10% with 4 out of 40 rats dying prematurely. The MABP was measured before the ischemic insult and immediately after. Prior to ischemia the MABP was 107.5±18.4 mmHg, during the ischemia it dropped to an average of 39.3±1.2 mmHg, while after ischemia it was 121.5±18.3 mmHg. Thus the MABP returned to its normal range. Other parameters such as pH, pCO_2_, pO_2_, and temperature were also monitored throughout the procedure and were all within the normal ranges (data not shown).

### Myograph

**Fig 1:**
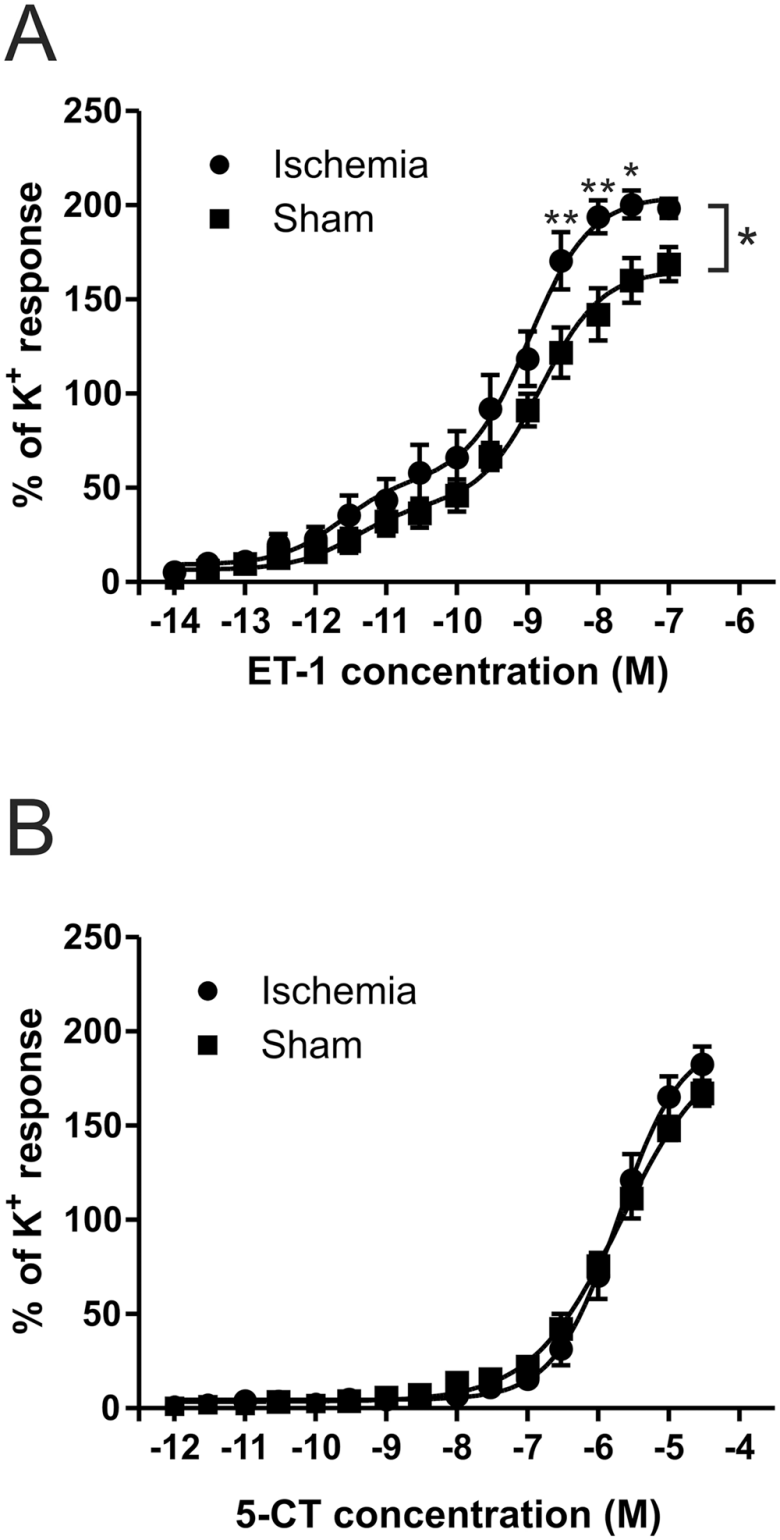
Increased endothelin-1 induced constriction after 48 hours. (**A**) Dose response curves for ET-1 on ischemic and sham ophthalmic artery 48 hours after ischemia are significantly different (p<0.05) with a left shift in the EC_50_. Sham EC_50_ was 1.56nM (pEC_50_ 8.810±0.12M) and for ischemia it was 1.07nM (pEC_50_ 8.97±0.13M). (**B**) 5-CT response curves are identical with EC_50_ 1.91μM (pEC_50_ 5.72±0.08M) for ischemia and EC_50_ 2.09μM (pEC_50_ 5.68±0.11 M) for sham.

Firstly, we aimed to investigate if there was indeed an increase of contractile function in the ophthalmic artery after global cerebral ischemia, similar to what we have observed in the brain after 48 hours. The concentration-response curves for ET-1 were significantly different 48 hours after ischemia (p<0.05), with a weak leftward shift in the EC_50_ in the ischemic animals, with an EC_50_ of 1.07 nM (pEC_50_ 8.97±0.13 M) for the ischemic animals and an EC_50_ of 1.56 nM (pEC_50_ 8.81±0.12 M) for sham operated animals (Fig 1). There was a significant increase in E_max_ (p<0.01) which shows a significant increase of ET-1 mediated vasoconstriction (Fig 1). However, the concentration-response curves for 5-CT were identical between both ischemic and sham operated animals with EC_50_ values of 1.91μM (pEC_50_ 5.72±0.08 M) and 2.09 μM (pEC_50_ 5.68±0.11 M) respectively (Fig 1). This suggests no difference in 5-HT receptor regulation between the two groups 48 hours post ischemia.

### Histology and Immunohistochemistry

To further investigate the retinal changes, we stained retinal cross sections using hematoxylin and eosin. We detected no noticeable differences in retinal thickness, cell density, or over-all morphology between the groups (data not shown).

**Fig 2:**
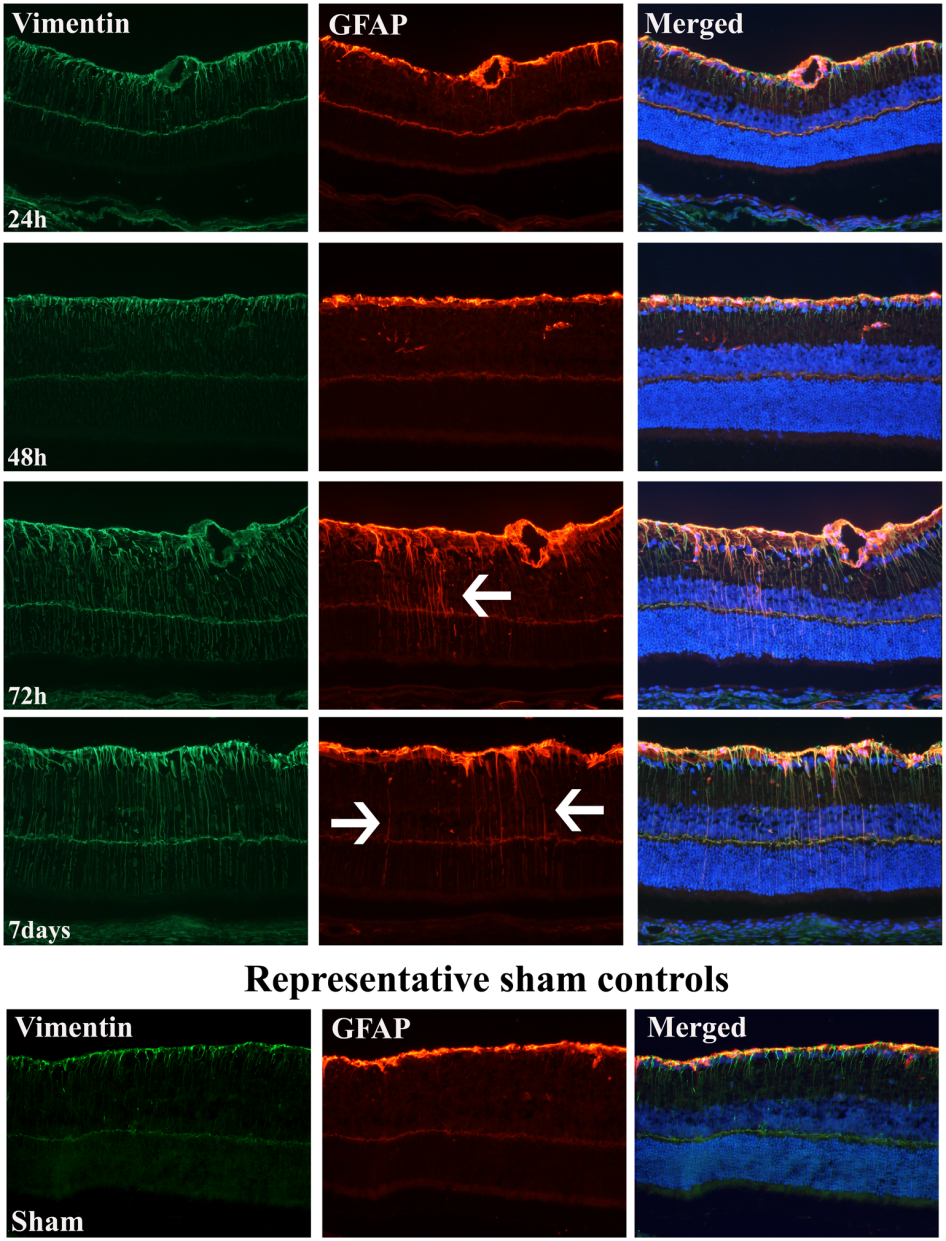
Increased GFAP and vimentin expression 72 hours and 7 days post ischemia. Increased GFAP and vimentin expression 72 hour and 7 days after transient global ischemia (1 day n = 4, 48h n = 8, 72h n = 4, 7 day n = 8). Arrows highlight GFAP positive staining spanning throughout the retina. Even though there was an increase in vasoconstriction on the ophthalmic artery at 48 hours, it took 72 hours for the retina to show signs of gliosis through Müller cell markers GFAP and vimentin upregulation. Müller cells were still active at 7 days after ischemia.

GFAP and vimentin, which are markers of Müller cell activation, were clearly upregulated for the first time 72h after ischemia. This upregulation was maintained up to 7 days post ischemia with strong immunoreactivity spanning throughout the entire retina, characteristic of Müller cells [10]. The earlier time points, 24 hours and 48 hours post ischemia revealed only immunoreactivity along the nerve fiber layer which coincides with previous observations during normal physiological conditions [10]. Furthermore, the majority of GFAP and vimentin immunopositivity seem to co-localize, emphasizing that they are expressed in the same Müller cell projections spanning through the retina (Fig 2). GFAP positive staining in ganglion cell layer and nerve fiber layer is likely attributed to GFAP positive astrocytes. Two different GFAP antibodies were examined and they resulted in identical patterns of staining.

### ERG

To investigate retinal function, ERG was performed and 3 days after surgery, both inner and outer retinal cell responses were compromised by ischemia.

**Fig 3:**
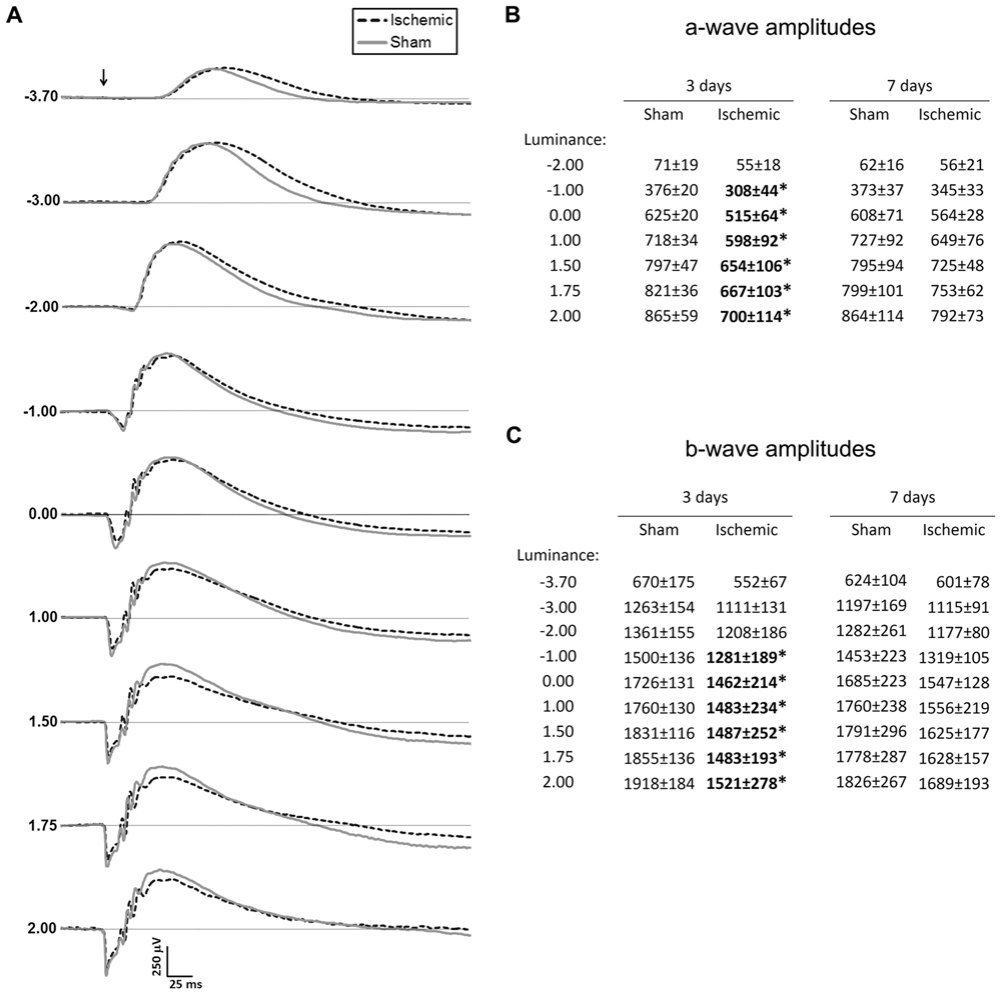
Representative Scotopic ERG response curves with average a and b-wave amplitudes. (**A**) Scotopic ERG responses representative of each experimental group to stimuli of increasing luminance on day 3. (**B**) Average a-wave amplitudes at day 3 and 7 after surgery. (**C**) Average b-wave amplitudes at day 3 and 7 after surgery. Arrow indicates time of stimulus; unit for luminance is log cd*s/m^2^; unit for amplitudes is μV; * denotes p<0.05.

Photoreceptor activity is reflected by the ERG a-wave. At 3 days after surgery, a-wave amplitudes of ischemic rats (n = 6) were approximately 20% lower than those of sham operated rats (n = 6) for luminances of -1.0 to 2.0 log cd*s/m^2^ (p<0.05; Fig 3A and 3B). Although dependent upon the a-wave, the magnitude of the b-wave reflects ON-bipolar cell activity and at this time point, b-wave amplitudes of ischemic rats (n = 6) were also approximately 20% lower than those of sham operated rats (n = 6) for luminances of -1.0 to 2.0 log cd*s/m^2^ (p<0.05; Fig 3A and 3C). Seven days after surgery, a statistically significant difference for either a- or b-wave amplitudes was not found (p>0.05; Fig 3). No differences in peak latencies were seen at either time point (not shown).

**Fig 4:**
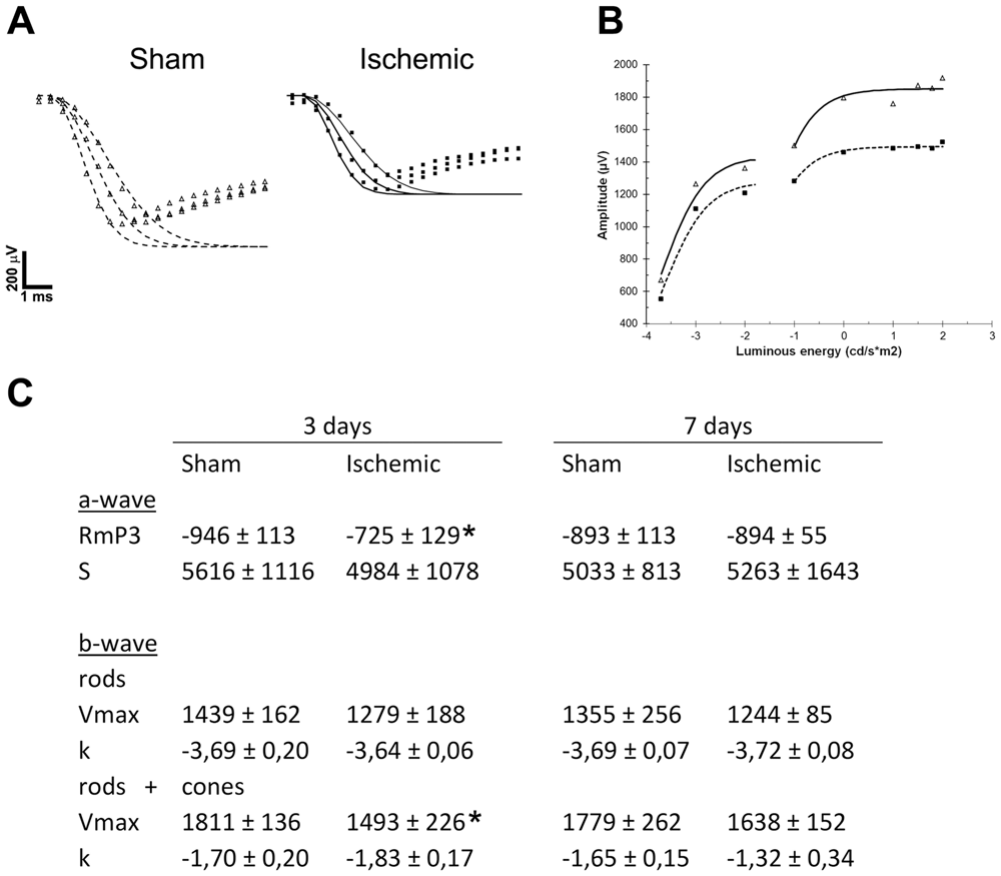
The falling phase of a-wave, Naka-Rushton modelling, and a representation plotted parameters of sham and ischemic rats. (**A**) The falling phase of a-wave responses to stimuli of 1.5, 1.75 and 2.0 log cd*s/m^2^ were modelled for both sham (unfilled triangles) and ischemic animals (filled squares). (**B**) Average b-wave responses at day 3 for sham operated (unfilled triangles) and ischemic (filled squares) are plotted here against luminous intensity. These responses were modelled using a Naka-Rushton function (dotted and continuous lines, respectively). (**C**) Optimization of the parameters for these functions (A, B) were made and averaged for both experimental groups, shown here for both 3 and 7 days after operation. Unit for S is log m^2^*cd^-1^*s^-3^; unit for k is log cd*s/m^2^; unit for *Rm*_*P3*_ and *Vmax* is μV; * indicates p<0.05.

Mathematical modelling of responses across various luminances can be useful for further analysis of retinal function. Using a model similar to that formulated by Hood and Birch (1990) for photoreceptor responses to luminous energies of 1.5, 1.75 and 2.0 log cd*s/m^2^, the saturated response amplitude, *Rm*_*P3*_, was found to be significantly reduced in ischemic animals 3 days after surgery, but not on day 7 (Fig 4A and 4C). No differences were found in sensitivity (*S*) on either day (Fig 4C). The b-wave amplitudes were plotted against luminous energy and fitted using a Naka Rushton function (Fig 4B). The saturated response amplitude, *Vmax*, for the higher luminous energy branch (mixed rod and cone responses) was lower in ischemic animals as compared to sham operated rats on day 3, but not on day 7 and not for the lower branches (Fig 4C). No differences were found for the semisaturation constant, *k* (Fig 4C).

**Fig 5:**
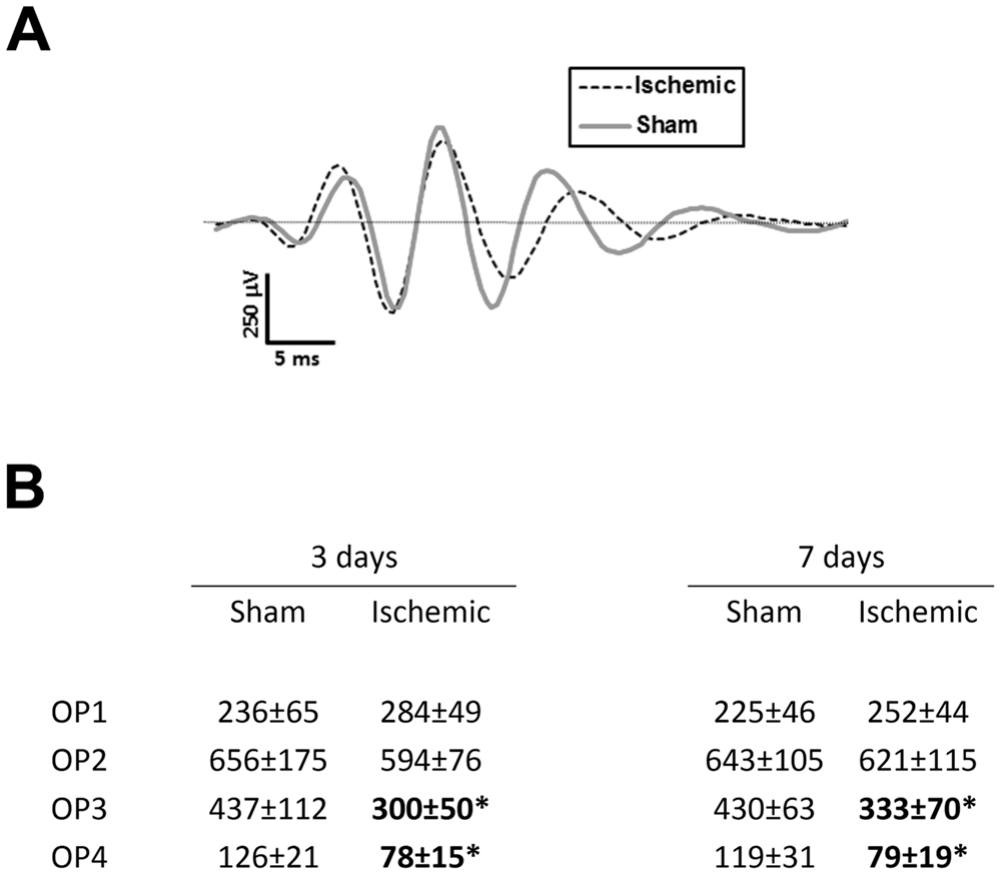
Oscillatory potential wavelets and average amplitudes or representative individuals from both sham and ischemic groups. (**A**) OP wavelets isolated from ERG traces (luminance: 2.0 log cd*s/m^2^) from one animal which is representative of each experimental group on day 3. (**B**) Average OP amplitudes for each experimental group at 3 and 7 days after surgery. Unit for amplitudes is μV; * denotes p<0.05.

Proximal retinal cell (possibly amacrine cell) activity is considered to give rise to the OPs on the rising face of the b-wave. These wavelets can be isolated by applying a band-pass filter and, when overlapped, differences between traces from the two experimental groups can be seen (Fig 5A). At day 3, the amplitudes of the third and fourth wavelets (OP3 and OP4) of ischemic rats were, respectively 30% and 40% lower than those of sham operated rats (p<0.05; Fig 5B). Seven days after surgery, OP3 and OP4 amplitudes were, respectively, 20% and 35% lower in ischemic rats (p<0.05; Fig 5B). No differences in wavelet latencies were seen at either time point (not shown).

**Fig 6:**
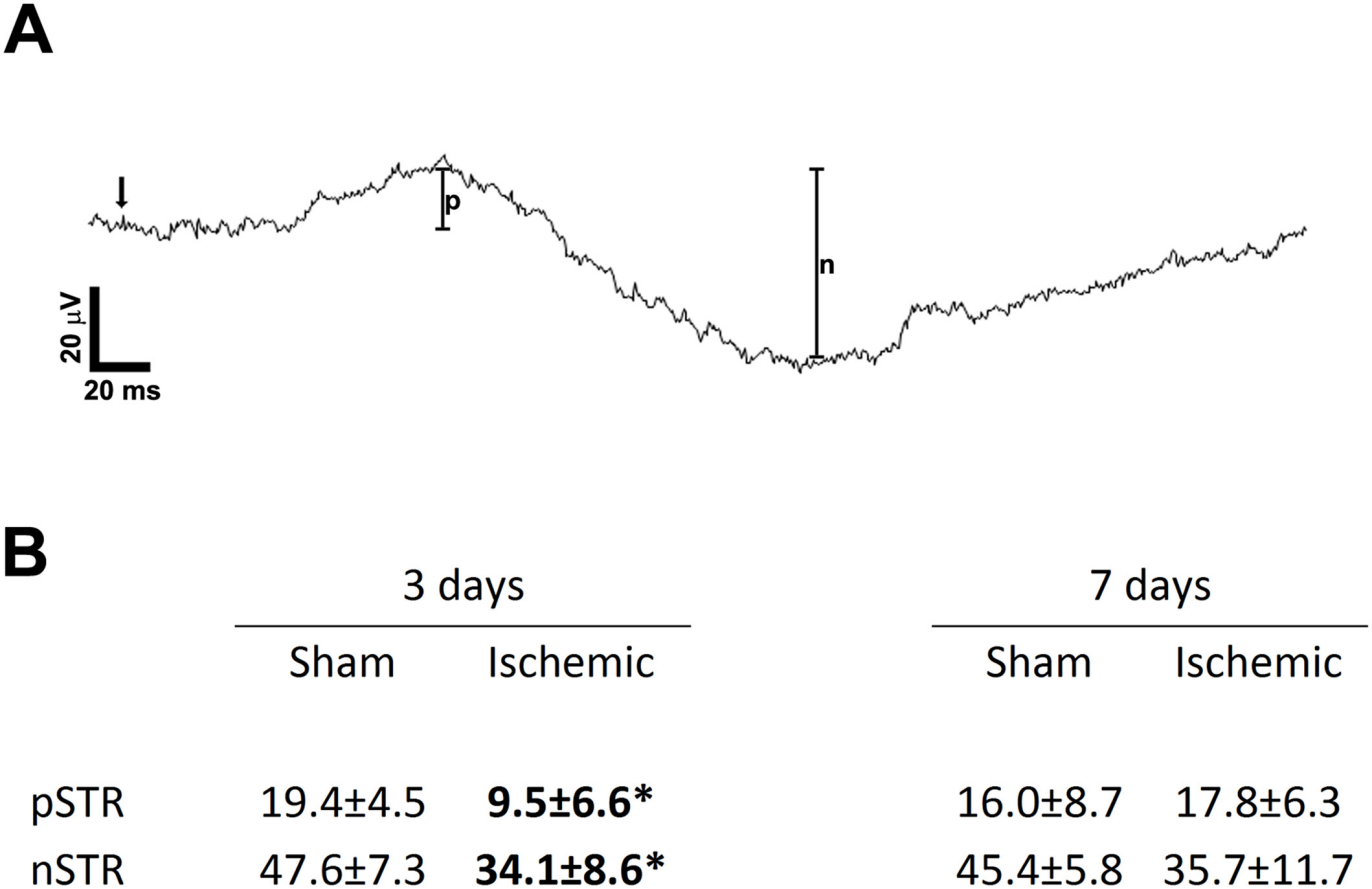
STR responses with average amplitudes of both sham and ischemic animals at 3 and 7 days post ischemia. (**A**) The amplitude of the pSTR was measured from baseline to the peak of the first positive potential (denoted by p) in response to a stimulus of -5.8 log cd*s/m^2^. The amplitude of the nSTR was measured from this peak to the trough of the following negative potential (denoted by n). (**B**) Average pSTR and nSTR amplitudes for each experimental group at days 3 and 7 after surgery. Arrow indicates time of stimulus; unit for amplitudes is μV; * denotes p<0.05.

The positive and negative scotopic threshold responses (STR; Fig 6A) are believed to mainly arise from the ganglion cells. In this study, the positive STR (pSTR) amplitudes of ischemic rats on day 3 were approximately 50% those of sham operated rats (n = 6; p<0.05; Fig 6B). The negative STR (nSTR) amplitudes of ischemic rats were also about 30% lower than those of sham operated rats on day 3 (n = 6; p<0.05; Fig 6B). On day 7, the amplitudes of the pSTR were similar in both groups (Fig 6B) and while the nSTR amplitudes of ischemic rats were still lower, this difference was not statistically significant (Fig 6B). No differences in peak latencies were seen at either time point (not shown).

## Discussion

We hypothesized that there would be vascular changes occurring after the ischemic insult which could cause or contribute to delayed damage to the retina. Here we are shedding new light on the link between cerebral and ophthalmic vasculature within the context of global cerebral ischemia. We discovered, for the first time, that vasocontractility of the ophthalmic artery is significantly increased 48 hours after global ischemia, thus it is believed to reduce blood flow to the eye long after the original ischemic insult [22]. We observe retinal damage occurring at 72 hours after the ischemic insult. Therefore we propose that this is caused by changes in the retinal vasculature, suggesting that they are an important factor in fully understanding retinal ischemia.

Retinal ischemia has been investigated for decades with a variety of methods. However, compared to the brain the retina has an innate resistance to ischemic insult. Retinal ischemia can be induced for up to 120 minutes with both ocular pressure increase or with vascular occlusion [23, 24], leading to dramatic effects 48 hours after ischemia. Though generally, a minimum of 30 minute complete ischemia is needed to elicit lasting and reproducible damage in the rat retina reviewed by Osborne and colleagues [25], and it has been shown that retinal ischemia is an ongoing process which is highly time dependent [26]. A recent study by Zhao and colleagues found a functional recovery after a 17 minute global cerebral ischemia [27]. However, our data on cerebral vasculature shows an upregulation of vascular contractile receptors which first occurs 48 hours after the ischemia [14]. We therefore propose that vascular changes can cause a second delayed ischemia and, crucially, even a shorter global ischemic insult of 15 minutes is sufficient to elicit delayed damage to the retina. Thus, judging from our results, we believe that the vasculature and the exuberated damage induced by receptor upregulation should be an important aspect in considering clinical targets for retinal ischemia.

Interestingly, our results show a similar increase of ET-1 mediated vasoconstriction occurring in the ophthalmic artery as observed in the cerebral arteries at 48 hours after ischemia [14]. The exact changes of ET-1 receptor expression in the ophthalmic artery was not evaluated in this study. However, in cerebral arteries ET_B_ expression in the smooth muscle cells of vasculature causes a significant portion of the observed increased vasoconstriction [6]. The exact expression of ET-1 receptors in the rat ophthalmic artery will be an aim of future studies. Still, the increased vasoconstriction which is determined after ischemia in this current study, could potentially lead to an increased ischemic damage to the retina. Another related aspect to be considered is the endothelium of the ophthalmic artery. Prior to the experimental procedure it was shown to be intact (data not shown). Additionally, the endothelial cells do not have an influence on the contractile response when receptor upregulation is investigated [28]. Nonetheless, cerebral and ophthalmic arteries do not seem to follow identical pathological patterns, as cerebral vessels also exhibited a strong 5-HT_1B_ receptor mediated vasoconstriction [14]. This would suggest that even though 5-HT receptors are involved in cerebral vascular response to ischemia, they are not affected by global cerebral ischemia in the ophthalmic artery.

After acute ischemic stroke, the level of ET-1 in blood plasma is increased up to four times its normal level [29]. The vasoconstrictive property of ET-1 is so strong that it is used as a noninvasive method to induce local ischemia, by injecting ET-1 into the eye under the conjunctiva causing sudden and complete constriction of retinal vasculature [30]. Further, ET-1 has been found to be active within the eye during ischemic conditions such as elevated intraocular pressure models. Here, ET_B_ has been found to decrease retinal ganglion cell survival during glaucoma, suggesting that an ET-1 antagonist may be of therapeutic value [31]. Thus, with our results in mind, ET-1 may in addition decrease blood flow not only through the ophthalmic artery, but also in the retinal vasculature, as well as having a detrimental effect on the survival of retinal ganglion cells. However, ischemic damage has been of temporary character on the retina after vascular ischemia such as global or middle cerebral artery occlusion [24, 27]. However these studies did not continue past the timespan where we observed the vascular changes occur.

GFAP has been revealed to be an extremely sensitive and non-specific indication of retinal damage and the initiation of gliosis, as indicated by its upregulation in the retina in: glaucoma, ischemia- reperfusion, retinal detachment, diabetic retinopathy, inflammation, and proliferative retinopathies [9]. Thus, GFAP can be seen as a universal stress indicator in the retina. Vimentin, also an intermediate filament, is commonly co-expressed in the retina with GFAP. Both proteins act as stabilizers for Müller cell processes and are involved in the signal transduction cascades essential in reactive gliosis [10]. Within ischemia/reperfusion injuries glutamate toxicity and VEGF induced neovascularization are two key players that affect the function of the retina [9]. Glutamate has been shown to be accumulated in the neural processes disrupting the ionic balance [25, 32] and leading to excitotoxicity. Furthermore, once a shortage of blood flow is detected in the eye VEGF promotes the formation of new vasculature. However, this vasculature is not included in the retinal blood barrier and thus lead to leakage and faulty vascularization, leading to reduced vision [25]. Thus an upregulation of GFAP and vimentin suggests that structure and/or function of the retina has been compromised after 15 minutes of global cerebral ischemia.

Functional ERG results at 72 hours showed a significant reduction in the a-wave and b-wave of ischemic animals. The sensitivity parameters were not altered in either of these components (*S* and *k*, respectively), suggesting that transduction was unaffected. Retinal function, however, was affected as modelling the responses to the higher luminous energies showed that the saturated response amplitudes for both (*Rm*_*P3*_ and *Vmax*, respectively) were lower in ischemic rats. Oscillatory potentials of the animals were also significantly reduced. Finally, the scotopic threshold response (STR) primarily reflecting ganglion cell activity [33] was also decreased at 72 hours, indicating a complete reduction in retinal function. However, the reduced function of the retina was not permanent. Even though there were still signs of gliosis throughout the retina at 7 days, the a-wave, b-wave, and STR responses had recovered as significant differences between sham and ischemic animals were no longer detectable. The oscillatory potentials, however, did not recover completely by day 7.

A recent study by Zhao and colleagues [27] showed that the retina recovered almost completely following a 17 minute global ischemic insult and reperfusion at 48 hours. Here we demonstrate an increased contractile response in the ophthalmic artery 48 hours after global cerebral ischemia, and thus a potential second round of reduced blood flow and ischemia. Interestingly, the increase in Müller cell activation was only observed after the increased contractility demonstrated in the ophthalmic artery. Therefore, the ophthalmic artery may be a valid and novel therapeutic target especially for longer ischemic episodes such as those following middle cerebral artery occlusions. Further studies are needed to evaluate the ophthalmic arteries vasoactive significance of the ophthalmic artery in other ischemic conditions.

## Conclusion

In conclusion, this study shows that there is a similar response between the cerebral and the ophthalmic vasculature after a global ischemic insult. Myograph data shows an increase of ET-1 mediated vasoconstriction 48 hours after global ischemia. This increase of contractility may exacerbate retinal damage after ischemia, as seen by the increase of GFAP and vimentin along with decreased retinal function 72 hours post ischemia. No difference in GFAP or vimentin immunopositivity was observed 24 to 48 hours between sham operated and ischemic animals. These data suggest that decreased blood flow in the ophthalmic artery might play a role in ischemic damage, and that retinal ischemic changes should be monitored for longer than 48 hours to detect delayed damage that might follow the vascular upregulation.
